# Baseline characteristics of patients from the Brazilian Severe Asthma Registry: the REBRAG study

**DOI:** 10.36416/1806-3756/e20240427

**Published:** 2025-07-22

**Authors:** Paulo Marcio Pitrez, Débora Carla Chong-Silva, Faradiba Sarquis Serpa, Adelmir Souza-Machado, Adalberto Sperb Rubin, Amanda da Rocha Oliveira Cardoso, Adyléia Aparecida Dalbo Contrera Toro, Laura Maria de Lima Belizario Facury Lasmar, Daniela Cavalet Blanco, Luciana de Freitas Velloso Monte, Marina Andrade Lima, José Gustavo Barian Romaldini, Eduardo Costa de Freitas Silva, Kamila Ticiana Dias Ferreira, Alvaro Augusto Souza da Cruz, Marcia Margaret Menezes Pizzichini, REBRAG group

**Affiliations:** 1. Hospital Santa Casa de Porto Alegre, Pavilhão Pereira Filho, Porto Alegre (RS) Brasil.; 2. Complexo Hospital de Clínicas da Universidade Federal do Paraná, Curitiba (PR) Brasil.; 3. Hospital Santa Casa de Misericórdia de Vitória, Centro de Referência em Asma Grave, Vitória (ES) Brasil.; 4. Instituto de Ciências da Saúde, Universidade Federal da Bahia, Salvador (BA) Brasil.; 5. Hospital das Clínicas da Universidade Federal de Goiás, Goiânia (GO) Brasil.; 6. Centro de Investigação em Pediatria, Universidade Estadual de Campinas, Campinas (SP) Brasil.; 7. Centro Multidisciplinar para Pacientes com Asma de Difícil controle - CEMAD - Hospital das Clínicas - HCUFMG/Ebserh - Faculdade de Medicina, Universidade Federal de Minas Gerais, Belo Horizonte (MG) Brasil.; 8. Pontifícia Universidade Católica do Rio Grande do Sul, Porto Alegre (RS) Brasil.; 9. Hospital da Criança de Brasília José Alencar, Brasília (DF) Brasil.; 10. Hospital Dia do Pulmão, Blumenau (SC) Brasil.; 11. Hospital Santa Casa de Misericórdia de São Paulo, São Paulo (SP) Brasil.; 12. Serviço de Alergia e Imunologia, Universidade Estadual do Rio de Janeiro, Rio de Janeiro (RJ) Brasil.; 13. Hospital das Clínidas da Universidade Federal de Pernambuco, Recife (PE) Brasil.; 14. Programa de Pós-Graduação em Medicina e Saúde e Fundação ProAR, Faculdade de Medicina da Bahia, Universidade Federal da Bahia, Salvador (BA) Brasil.; 15. Programa de Pós-Graduação em Ciências Médicas, Universidade Federal de Santa Catarina, Florianópolis (SC) Brasil.

**Keywords:** Asthma, Phenotype, Biological products

## Abstract

**Objective:**

To describe the impact of severe asthma in a real-life cohort in Brazil, reporting on baseline clinical characteristics, access to treatment, and clinical remission under treatment with biologics.

**Methods:**

Severe asthma patients > 6 years of age were recruited from 23 centers in Brazil. Data on clinical characteristics, lung function, biomarkers, prescribed therapies, and clinical remission under treatment were collected at the baseline visit.

**Results:**

A total of 417 patients were recruited. Of the 162 adult patients, 71% had a history of hospitalization, with 31% having experienced more than two severe exacerbations in the last 12 months and 6% having experienced cardiopulmonary arrest. Allergic and eosinophilic phenotypes were the most common phenotypes in all age groups, with the T2-low phenotype being observed in 10% of the pediatric patients and in 20% of the adult patients. Only 10% of the adult patients and 1% of the pediatric patients were receiving maintenance oral corticosteroids, whereas 41% of the adult patients were under treatment with biologics, with clinical remission being achieved in 20%.

**Conclusions:**

Severe asthma in Brazil still results in a high disease burden, with less than half of the patients receiving treatment with biologics and clinical remission being achieved in a subgroup of patients treated with biologics for more than 12 months. Achieving disease control remains a major clinical and health care challenge, requiring further actions from specialists and health care providers, as well as additional studies.

## INTRODUCTION

Severe asthma is defined as asthma requiring treatment with high doses of inhaled corticosteroids (ICS) and a second controller medication, as well as optimal inhaler technique, optimal treatment adherence, and effective control of comorbidities; patients with severe asthma constitute 3-4% of the total asthma population.^1^ ^,^ ^2^ Patients with severe asthma experience the highest rates of morbidity, an increased risk of hospitalization, and loss of lung function, placing a substantial financial burden on health care systems.^3^ ^-^ ^7^ In developing countries, the impact of severe asthma is even more challenging, which is due to the difficulty in establishing an accurate diagnosis, receiving specialist follow-up, and gaining access to high-cost therapies.^8^

Although there have been studies conducted at referral centers and providing local evidence on severe asthma in small patient samples, studies involving a robust number of participants and prospective follow-up are essential for understanding the impact of severe asthma, patient phenotypes, treatment responses (particularly to biologics), the ability to achieve remission with new targeted therapies, and long-term prognosis. As is the case with the International Severe Asthma Registry (ISAR),^9^ real-world data from different countries, including Brazil, are important to addressing regional questions regarding phenotypic profiles, access to treatments, and responses to high-cost therapies. In this context, the *Registro Brasileiro de Asma Grave* (REBRAG, Brazilian Severe Asthma Registry), established in 2021, stands out as a multicenter cohort of pediatric and adult patients with severe asthma, with an annual collection of real-world clinical data. The present study was the first by the REBRAG group, and the objective of the study was to describe the characteristics of severe asthma patients at baseline (including demographic data, comorbidities, level of disease control, level of disease impact, and phenotypic profile) and the therapies used, reporting on the proportion of patients who achieved clinical remission with those therapies. 

## METHODS

### Study design

The REBRAG study was a nationwide, multicenter, prospective cohort registry study. Real-world patient data were collected via electronic medical record review at 23 severe asthma centers in Brazil. The baseline visit was the entry visit, and the same parameters were collected annually. In the present study, we present the results for the 2021-2023 period. 

### Patients

The inclusion criteria were as follows: 1) ≥ 6 years of age; 2) having a history of symptoms suggestive of asthma; 3) meeting the GINA criteria for severe asthma; 4) having a history of asthma symptoms; 5) having a confirmed diagnosis of asthma based on the presence of reversible expiratory airflow limitation with the use of albuterol; 6) having used high-dose ICS with at least one long-acting β_2_ agonist (LABA) for more than 6 months, with or without oral corticosteroids; and 7) having received optimal management (including good treatment adherence, correct inhaler technique, and management of treatable comorbidities) for at least 6 months of follow-up at our referral center. Patients with other chronic pulmonary diseases, cognitive impairment, neurological disorders, or immunodeficiencies were excluded. 

### Ethics

The present study was approved by the research ethics committees of all REBRAG centers, and all participating patients and/or their legal guardians gave written informed consent. 

### Data collection, tests, and procedures

Data were entered into an encrypted web-based online registry system with integrated plausibility checks within the database and a random sample of records regularly monitored at each participating center. The collected data included the following: demographic data and medical history (including symptoms, medications, and comorbidities); Asthma Control Test (ACT) or Childhood ACT scores; Asthma Quality of Life Questionnaire or Pediatric Asthma Quality of Life Questionnaire scores; use of rescue medication; exacerbations in the past 12 months; pulmonary function (FEV_1_, FVC, and PEF); and biomarkers (complete blood count, total IgE, aeroallergen sensitization, and fractional exhaled nitric oxide). Clinical phenotyping was considered complete when patients had undergone eosinophil count, determination of total serum IgE levels, and aeroallergen sensitization testing. 

Airflow limitation was classified in accordance with the severity of respiratory obstruction. Mild airflow limitation was defined as an FEV_1_ ≥ 60% of the predicted value up to the lower limit of normal; moderate airflow limitation was defined as an FEV_1_ of 41-59% of the predicted value; and severe airflow limitation was defined as an FEV_1_ of ≤ 40% of the predicted value. Reversibility was defined as a ≥ 12% increase in FEV_1_ after bronchodilator administration.^10^

Severe asthma phenotypes were classified as allergic asthma, eosinophilic asthma, or T2-low asthma, with cases of overlapping allergic and eosinophilic asthma also being considered. The criteria for diagnosing the phenotypes were as follows: allergic asthma-at least one positive skin prick test or blood test for aeroallergens; eosinophilic asthma-blood eosinophils ≥ 150 cells/mm^3^; and T2-low asthma-absence of aeroallergen sensitization and blood eosinophils of < 150 cells/mm^3^.^1^

Clinical remission was evaluated in patients in GINA treatment step 5 and was defined as no exacerbations or use of oral corticosteroids in the past 12 months and an ACT or Childhood ACT score > 20 on the day of the baseline visit, for at least 12 months after initiation of treatment with a biologic agent or triple therapy.^11^

### Statistical analysis

Descriptive statistics were created for the sociodemographic and clinical characteristics of the patients, being presented as mean and standard deviation; median and interquartile range; or proportions. Numerical variables were presented as mean and standard deviation or median and interquartile range, depending on the distribution of the variables. Categorical variables included frequency tables with counts and percentages. A 95% confidence interval was used when applicable. All tests were two-tailed, and the level of significance was set at 5%. All statistical analyses were performed with RStudio, version 4.4.2 (RStudio, Inc., Boston, MA, USA). 

## RESULTS

A total of 417 patients were included in the present study. Of those, 146 (35%) were in the pediatric age group (< 18 years of age), 162 (38.9%) were in the 18- to 59-year age bracket, and the remaining 26.1% were ≥ 60 years of age. Approximately two thirds of the children were male, unlike the adult group, where women predominated. Slightly more than half of the participants were White. No participant < 18 years of age was a smoker, and the proportion of former smokers was higher with increasing age. Table 1 presents the characteristics of the study participants. With regard to the level of education, 50% had had 9 years of schooling, 5% were illiterate, and 96% used the public health care system exclusively. 

**Table 1:** Characteristics of severe asthma patients (n=417) and disease burden at baseline.^a^

	< 18 years of age (n = 146)	18-59 years of age (n = 162)	≥ 60 years of age (n = 109)
Age, years	12 [10-14]	47 [40-54]	67 [62-72]
Sex, male	86 (59)	45 (28)	30 (28)
White	92 (63)	87 (54)	64 (59)
Smoker			
Never smoker	146 (100)	138 (85)	72 (66)
Former smoker	0 (0)	22 (14)	33 (30)
Current smoker	0 (0)	2 (1)	4 (4)
Treatment			
ICS, µg/day*	500 [500-1,000]	600 [500-1,000]	500 [500-500]
Uncontrolled disease	67 (46)	107 (66)	57 (53)
Early-onset asthma, < 18 year of age	146 (100)	112 (72)	52 (50)
Age at onset of asthma, years	1 [0.5-3]	7 [1.91-20]	18 [5-35]
Age at asthma diagnosis, years	4 [2-6]	14 [5-30]	30 [8- 43.5]
> 2 severe exacerbations in < 12 months	49 (34)	51 (31)	28 (26)
Hospitalization			
History of hospitalization	109 (75)	114 (70)	75 (69)
Number of hospitalizations	6.0 [2-13]	10.0 [3-20]	10.0 [4-20]
ICU admissions	42 (29)	39 (24)	22 (20)
> 2 ICU admissions	6 (4.1)	12 (7.4)	8 (7.3)
Orotracheal intubation	14 (9.6)	23 (14)	14 (13)
Cardiopulmonary arrest	5 (3.4)	15 (9.3)	6 (5.5)

ICS: inhaled corticosteroids. ^a^Data expressed as n (%) or median [IQR]. *Budesonide-equivalent doses.

Of the 417 patients evaluated in the present study, 231 (55%) were diagnosed with uncontrolled asthma at the time of the first visit on the basis of their ACT scores (Table 1). Early-onset asthma was identified in 164 (61%) of the 271 patients ≥ 18 years of age. A total of 128 (31%) of the 417 patients included in the study reported more than two severe exacerbations in the past 12 months, with severe exacerbations being more prevalent in children (in 49 [34%] of a total of 146). A history of hospitalization was reported by 298 (71%) of the 417 patients included in the present study. Of those, 103 (25%) were admitted to the ICU. 

**Figure 1:**
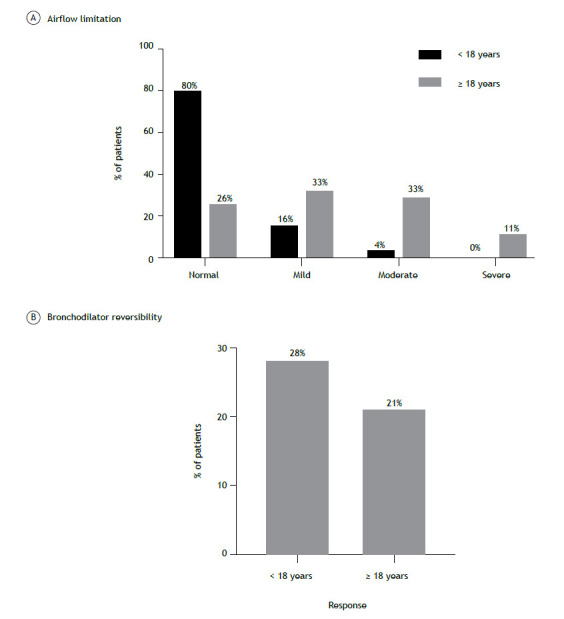
Airflow limitation (in A) and bronchodilator reversibility (in B) in pediatric and adult patients with severe asthma in Brazil, (n = 363).

Regarding lung function, 111 (80%) of the 138 patients < 18 years of age showed no evidence of obstructive ventilatory defect. Of the 225 patients ≥ 18 years of age, 59 (26%) showed no evidence of obstructive ventilatory defect (p < 0.01; Figure 1A). Reversibility of airflow limitation was observed in 38/135 patients < 18 years of age (28%), whereas, in patients ≥ 18 years of age, reversibility of airflow limitation was observed in 48/225 (21%; Figure 1B). When we analyzed FEV_1_ values that did not normalize after bronchodilator administration, we found that 183/225 (81%) of the adult patients showed no reversibility to normal lung function parameters. 

**Figure 2:**
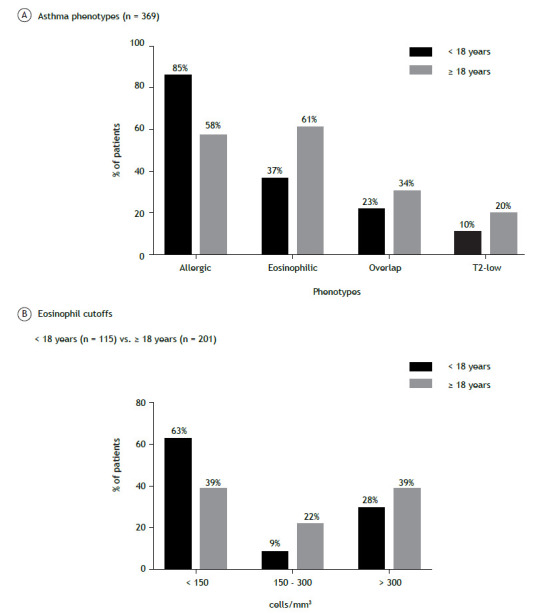
Asthma phenotypes (in A) and eosinophil cutoffs (in B) in pediatric and adult patients with severe asthma in Brazil.

Complete clinical phenotyping was performed in 369 (84%) of the 417 patients in the study sample. Among pediatric patients, allergic asthma was the predominant phenotype, whereas overlapping allergic and eosinophilic phenotypes predominated in adult patients. The T2-low phenotype was identified in 8 (10%) of the 82 pediatric patients tested and in 11 (20%) of the 68 adult patients tested. Figure 2 shows the distribution of phenotypes and blood eosinophil levels. 

**Figure 3:**
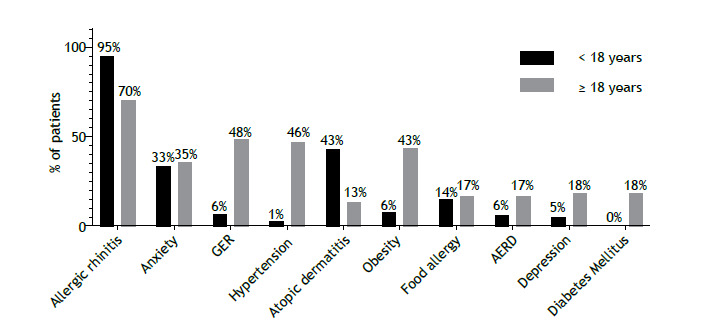
Comorbidities in pediatric and adult patients with severe asthma in Brazil (n = 417). GER: gastroesophageal reflux; and AERD: aspirin-exacerbated respiratory disease.

The most prevalent comorbidity in patients < 18 years of age and in those ≥ 18 years of age was allergic rhinitis, in 139/146 (95%) and 189/271 (70%), respectively. Other prevalent comorbidities in adults were anxiety, gastroesophageal reflux, obesity, and hypertension. In children, atopic dermatitis and anxiety were common comorbidities (Figure 3). 

**Figure 4:**
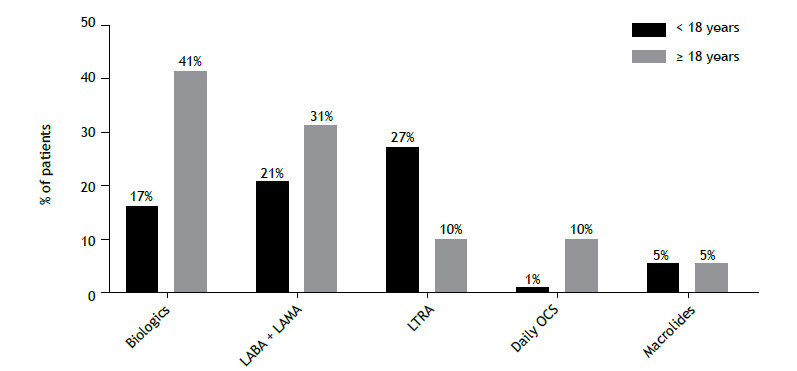
Therapies used in pediatric and adult patients with severe asthma in Brazil (n = 417). ICS: inhaled corticosteroid; LABA: long-acting β_2_ agonist; LAMA: long-acting muscarinic antagonist; LTRA: leukotriene receptor antagonist; and OCS: oral corticosteroid.

Regarding the therapies used, in the pediatric and adult patients, respectively, 21 and 31% were using triple therapy, and 17 and 40% were using biologics (Figure 4). Of the 23 patients < 18 years of age using biologics, 22 (96%) were using omalizumab and 1 (4%) were using mepolizumab, with a median duration of use of 21 months (IQR, 14-28 months). Of the 111 patients ≥ 18 years of age using biologics, 75 (68%) were using omalizumab, 28 (25%) mepolizumab, 5 (4%) benralizumab, and 3 (3%) dupilumab, with a median duration of use of 38 months (IQR, 8-89 months). One third of the pediatric patients in our sample were using leukotriene receptor antagonists, and only a few patients were under daily, long-term oral corticosteroid treatment (1% of the pediatric patients and 10% of the adult patients). 

**Figure 5:**
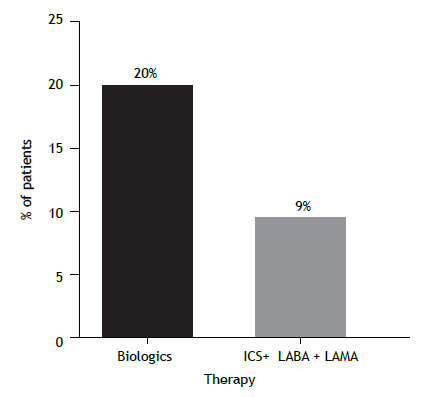
Clinical remission in severe asthma patients (≥ 18 years of age) by type of therapy (n = 99). ICS: inhaled corticosteroid; LABA: long-acting β_2_ agonist; and LAMA: long-acting muscarinic antagonist.

Clinical remission was observed in 13 (20%) of the 66 patients ≥ 18 years of age who used biologics. Of the 33 patients ≥ 18 years of age who used a combination of ICS, LABA, and a long-acting muscarinic antagonist, 3 (9%) achieved clinical remission (Figure 5). We were unable to evaluate clinical remission in the patients < 18 years of age because the number of patients available for analysis was low. 

## DISCUSSION

This was the first analysis of the REBRAG, based on data obtained at the first visit of patients (predominantly from the public health care system) and presenting a real-life overview of severe asthma in Brazil. The population of adult patients with severe asthma is predominantly female, unlike the pediatric population, which shows increased rates of obesity and physical inactivity. The predominance of adult women with severe asthma reflects national and international reports, as does the high prevalence of obesity and physical inactivity.^5^ ^,^ ^12^ Although severe asthma is more common in males than in females during childhood and adolescence, it becomes more common in females in adulthood, as observed in our study and as reported in previous studies.^5^ ^,^ ^12^

In the present study, the prevalence of smoking in adult patients was low (1%) in comparison with that of former smokers (14%). According to data from the 2019 Brazilian National Health Survey, the prevalence of smoking among adults in Brazil was approximately 12.6%.^13^ Our results show that patients with severe asthma in the country seem to avoid or quit smoking, which becomes a less significant risk factor for these patients. Our results also show that comorbidities are common, being consistent with ISAR data.^14^ Many comorbidities have a negative impact on disease management and should therefore always be evaluated in the diagnosis and management of severe asthma. The prevalence of nasal polyposis was low in our sample, possibly reflecting underdiagnosis as a result of limited access to specialized tests in public health care facilities, which accounted for 96% of the records analyzed in the present study. 

Despite being followed at referral centers in Brazil, many (66%) of the patients in our study had uncontrolled disease on the day of the first visit. This is consistent with data from international referral centers,^12^ highlighting the need for continuous reassessment of clinical approaches and access to therapies. In Brazil, disease control is achieved in only 12% of patients with asthma, regardless of its severity.^15^ Additionally, high rates of severe exacerbations in the past year, hospitalizations, and ICU admissions were observed, as well as many cases of cardiopulmonary arrest, with results similar to those of a study conducted over a decade ago at a referral center in Brazil.^5^ These data show that patients with severe asthma still experience high morbidity and an elevated risk of mortality. In this context, data from the Brazilian government show that asthma mortality has increased in recent years.^16^

Most (80%) of the pediatric patients in the present study had normal lung function, unlike the adult patients (26%). Additionally, lung function did not turn to normal values after bronchodilator administration in 81% of the adult patients in the present study. This might reflect the progressive, irreversible loss of lung function experienced by adult patients with severe asthma, being likely due to airway remodeling. Approximately 55% of the adult patients in the present study had early-onset asthma, being at a higher risk of developing persistent airway obstruction over time. Furthermore, we found that it may take 2 years to establish a diagnosis of asthma in children and 6 years to establish a diagnosis of asthma in adults, highlighting how the journey of patients with severe asthma is compromised right from the onset of their diagnosis. These findings show several factors that increase the risk for poor lung function over time in patients with severe asthma in Brazil. Studies have shown that severe asthma and allergic asthma are both risk factors for progressive, irreversible loss of lung function.^6^ ^,^ ^17^ ^,^ ^18^ Thus, studies including effective interventions for anti-T2 inflammatory activity early at the onset of the disease are needed to analyze their effect on preventing bronchial remodeling. 

The most prevalent phenotype in the present study was allergic asthma (in 66% of the study sample), the T2-low phenotype being the least common (in 19%). Allergic asthma predominated in children and adolescents (in 78%), whereas, in the adult population, the most common phenotype was eosinophilic asthma (in 61%). It is known that childhood-onset asthma is characterized by a more homogeneous phenotype, often associated with an allergic profile. In contrast, eosinophilic asthma is the most common phenotype in adults with severe asthma.^19^ ^,^ ^20^ We found that overlapping allergic and eosinophilic phenotypes are common in severe asthma patients, being observed in 60% of the children and in 61% of the adults in the present study. Patients with overlapping allergic and eosinophilic phenotypes have more than one option for specialists in decision-making regarding the choice of treatment with biologics, although other factors must be taken into consideration, including the presence of comorbidities, relying solely on the presence of biomarkers. 

When we analyzed the types of therapies that the patients in the present study were receiving, we found that less than 40% of the patients, regardless of age, were receiving treatment with biologics or triple therapy. In a report by the ISAR study group, 25% of adult patients with severe asthma worldwide were being treated with biologics.^21^ These results, along with the fact that many patients still had uncontrolled disease, suggest that patients are being undertreated, either because of misdiagnosis about severe asthma or because of a lack of access to GINA step 5 therapies. On the other hand, the fact that only 10% of the adults and 1% of the children in the present study were receiving long-term oral corticosteroid treatment shows the international trend to recommend this type of treatment as the last treatment option for severe asthma.^1^ Despite the most recent recommendations to restrict the use of leukotriene receptor antagonists because of their neuropsychiatric adverse effects, this class of medication is still frequently prescribed, particularly in children (27%).^22^

Another interesting finding of our study concerns clinical remission. We found that 20% of the patients treated with biologics and 9% of those receiving triple therapy achieved clinical remission 12 months after the onset of treatment. Clinical remission has been widely discussed in recent years,^11^ particularly with the emergence of biologic agents. Clinical remission achieved with new therapies has become a central goal for many diseases, including severe asthma, opening new pathways for the control of severe and complex diseases. Our initial results in this cross-sectional analysis show that clinical remission with triple therapy or biologics is a reality for many adult patients. Pulmonary function did not differ significantly between patients who achieved clinical remission and those who did not. Previous studies have shown clinical remission rates of 30-46% after 12 months of starting biologics in adult patients with severe asthma.^23^ ^-^ ^26^

Our study has some limitations, such as its cross-sectional nature, preventing us from presenting results regarding responses to therapies, prognostic risk factors, and lung function changes. Given the long-term follow-up nature of the REBRAG, future analyses have the potential to clarify these issues. Additionally, because our data come from real-life clinical evaluations, missing information poses a particular challenge, being mostly due to a lack of coverage or barriers to accessing certain tests through the public health care system. On the other hand, our study is valuable in that it shows real-life data from referral centers in Brazil, with broad representativeness across regions. Through the recruitment of patients from 23 centers across Brazil, our study shows a real-life profile of patients with severe asthma in the country and offers insights into the overall care of severe asthma patients. 

In conclusion, the initial results of the REBRAG study show that patients with severe asthma in Brazil, regardless of age, still experience high morbidity rates, with worsening pulmonary function in adulthood, often without significant reversibility in many patients. Achieving disease control remains a clinical and health care challenge, with the prospect of improvement through enhanced strategies for better access to higher-cost therapies (triple therapy and biologics). Finally, future results from the REBRAG study, with long-term longitudinal follow-up of participants, will potentially provide further insights into how to improve the quality of life and costs of patients with severe asthma in Brazil. 
